# Acinetobacter baumannii Causing Complex Parapneumonic Effusion in a Community-Living Immunocompetent Host: A Case Report

**DOI:** 10.7759/cureus.100179

**Published:** 2025-12-27

**Authors:** Dharani Abeyrathna, Lanka Wijekoon, Chalaka Kumarasinghe, Prasanna Weerawansa, Sisira Siribaddana

**Affiliations:** 1 Postgraduate Institute of Medicine, University of Colombo, Colombo, LKA; 2 Department of Medicine, Faculty of Medicine and Allied Sciences, Rajarata University of Sri Lanka, Anuradhapura, LKA; 3 Teaching Hospital Anuradhapura, Ministry of Health Sri Lanka, Anuradhapura, LKA

**Keywords:** carbapenem resistance, community-acquired pneumonia (cap), complex pleural effusion, pleural empyema, acinetobacter baumannii

## Abstract

Pleural effusion is a prevalent clinical condition; however, the occurrence of community-acquired *Acinetobacter* pneumoniae resulting in pleural effusion is infrequent. This case report describes a community-acquired complex pleural effusion due to *Acinetobacter baumannii*, which was successfully managed. A 56-year-old male patient presented to a local hospital with fever and right-sided pleuritic chest pain and was diagnosed with pleural effusion. Due to a lack of improvement after three days of intravenous meropenem antibiotics, the patient was transferred to a tertiary care hospital. Pleural aspiration confirmed a community-acquired *Acinetobacter baumannii*-infected complex atypical parapneumonic pleural effusion. The patient was treated with intravenous piperacillin-tazobactam, ciprofloxacin, and oral clindamycin antibiotics, along with pigtail catheter drainage, resulting in complete resolution of the condition. This report highlights the significance of early identification and effective management of atypical bacterial infections in pleural effusions.

## Introduction

Pleural effusion is a common complication of community-acquired pneumonia [1,2]. *Acinetobacter baumannii* is primarily recognised as a nosocomial pathogen that poses a significant threat to public health due to its propensity for antimicrobial resistance [3]. While carbapenem-resistant *Acinetobacter baumannii* (CRAB) has been classified by the WHO Bacterial Priority Pathogen List as a critically important pathogen in the highest priority category [4], community-acquired *Acinetobacter *infections can exhibit varying resistance patterns.

*Acinetobacter baumannii* infections acquired in hospital settings are frequently associated with extensive drug resistance and high mortality rates, ranking among the top five deadliest antibiotic-resistant pathogens worldwide. The organism is the leading cause of antimicrobial resistance (AMR)-related deaths in Southeast Asia, East Asia, and Oceania [4]. However, community-acquired *Acinetobacter* infections have distinct epidemiological and microbiological characteristics compared to their nosocomial counterparts.

Community-acquired *Acinetobacter* infections have been increasingly reported in Southeast Asia, Northern Australia, and Pacific islands, affecting both immunocompromised and immunocompetent individuals [2,5-7]. The higher prevalence of community-acquired *Acinetobacter* in specific geographical areas, particularly in tropical and subtropical regions, may be attributed to environmental factors and climatic conditions, including warm, humid climates, heavy rainfall, and soil/water exposure [2]. Established risk factors for community-acquired *Acinetobacter *infections include chronic alcoholism, diabetes mellitus, chronic obstructive pulmonary disease (COPD), current smoking, chronic kidney disease, and structural lung disease [5]. Unlike hospital-acquired strains, community-acquired isolates often demonstrate greater susceptibility to antimicrobial agents, though resistance patterns can vary significantly [5,8]. The burden of antimicrobial-resistant *Acinetobacter baumannii*, including carbapenem-resistant strains, remains substantial in South Asia [9].

Community-acquired *Acinetobacter* pneumonia complicated by pleural effusion is uncommon and may pose diagnostic and therapeutic challenges, particularly when the isolate is resistant to commonly used empirical antibiotics. This case report presents an unusual complex parapneumonic septated pleural effusion (a pleural effusion with features indicating infection, including low glucose, elevated lactate dehydrogenase (LDH), and septations requiring drainage) caused by community-acquired *Acinetobacter baumannii* with carbapenem resistance in an immunocompetent patient living in the community, highlighting the importance of culture-guided antimicrobial therapy even in community settings.

## Case presentation

A 56-year-old previously healthy man with no history of diabetes, malignancy, HIV infection (confirmed by negative serology), or immunosuppressive therapy presented with a four-day history of high-grade fever and right-sided pleuritic pain at a local hospital. The patient resided in a rural area of the North Central Province of Sri Lanka, a tropical region characterised by high humidity and temperatures. The patient had no occupational exposures to healthcare settings or known contact with hospitalised individuals. He had not taken antibiotics before the presentation. None of the family members had respiratory infections or any chronic illnesses. He also complained of chest pain, shortness of breath at rest, and a productive cough with yellow sputum. He was then transferred to a tertiary care hospital on the seventh day of illness due to clinical deterioration despite receiving intravenous meropenem for three days.

**Figure 1:**
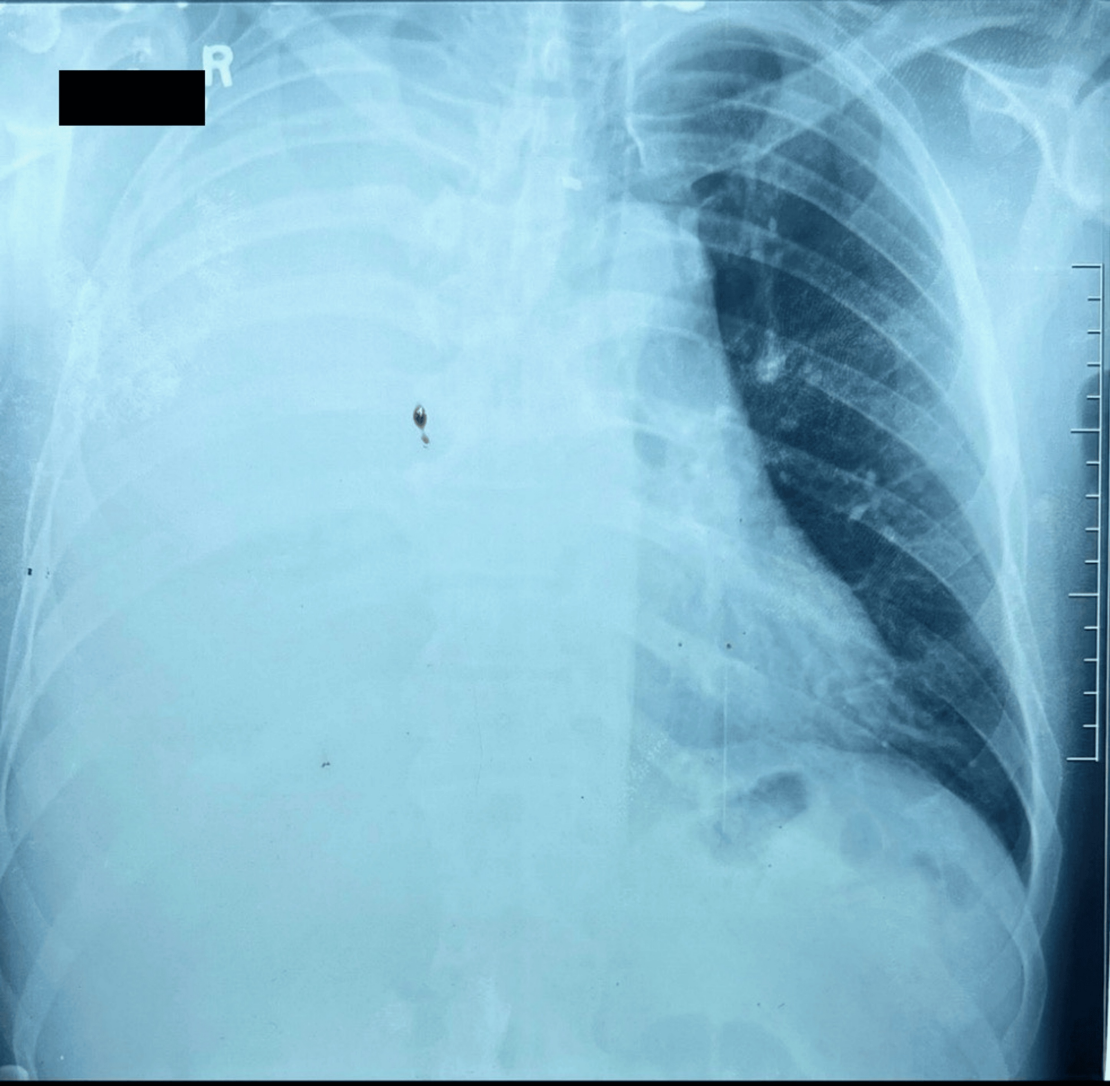
Chest radiograph on day seven demonstrated a right sided large pleural effusion

On presentation to the tertiary care hospital, he was febrile and tachypneic with a respiratory rate of 30 breaths per minute, and his blood pressure was 149/79 mmHg. Pulse oximeter reading displayed 99% saturation while breathing room air. Examination demonstrated stony dullness on percussion, reduced breath sounds with bronchial breathing in the right lower zone. The initial chest radiograph at the local hospital revealed right lower zone consolidation with mild pleural effusion; a repeat radiograph on day seven demonstrated a large pleural effusion (Figure 1).

**Figure 2:**
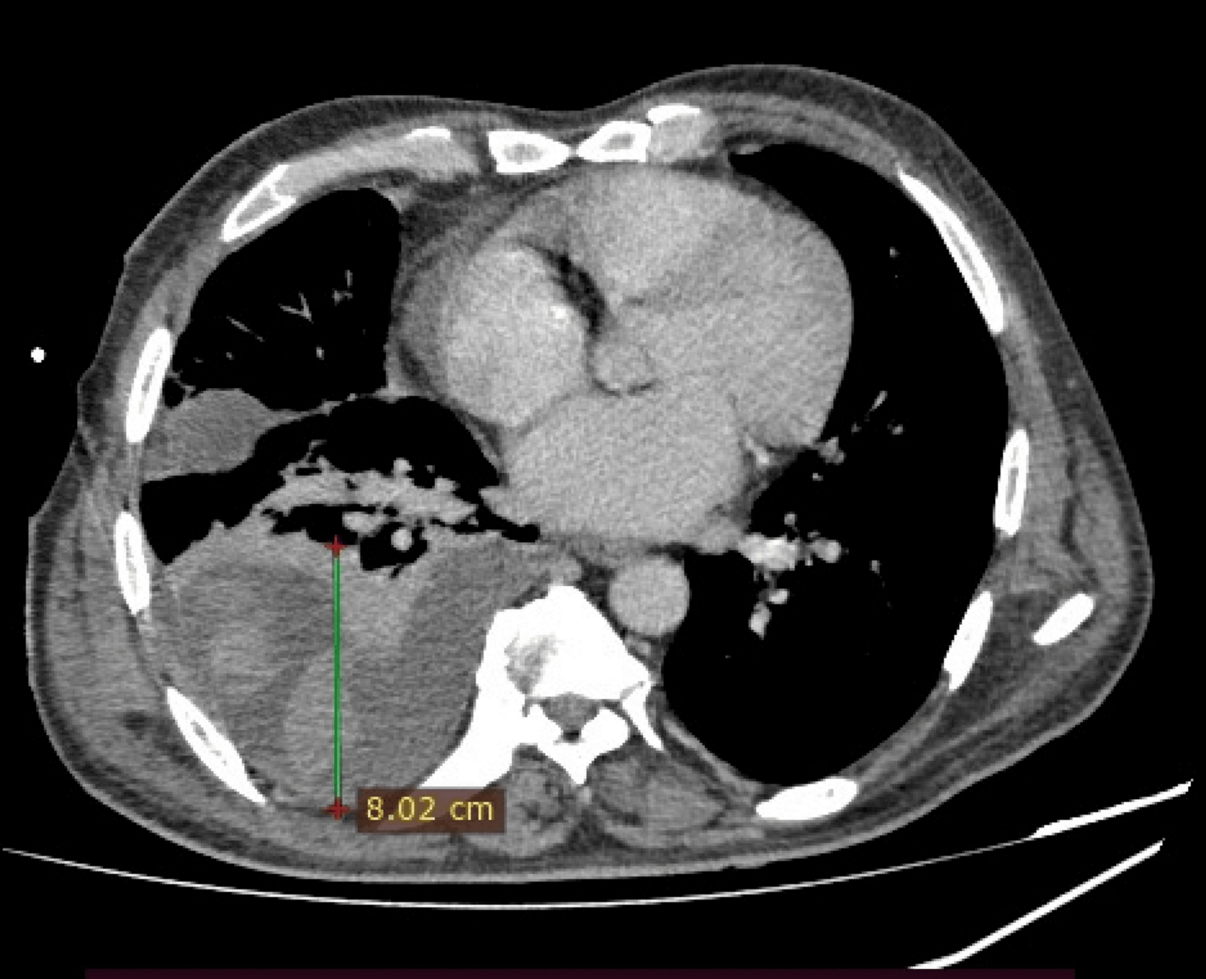
CECT Chest showing right sided complex pleural effusion

Contrast-enhanced computed tomography (CECT) of the chest revealed atelectatic alterations in the right lung and a right-sided moderately septated complex pleural effusion with approximately 8 cm maximum separation of the parietal and visceral pleura (Figure 2).

The sputum culture grew *Coliform* species with pus cells 10-25 and epithelial cells <10 per low-power field, and was sensitive to amikacin, cefuroxime, ciprofloxacin, co-amoxiclav, and gentamycin. Given the low pus cell count, this was considered likely oropharyngeal contamination rather than a true pathogen.

All pleural aspiration attempts were performed under strict aseptic technique with sterile gloves, drapes, and skin preparation using chlorhexidine solution. Blind pleural fluid aspiration without sonar image guidance was initially difficult, with three failed attempts. However, straw-coloured pleural fluid was successfully drained on the tenth day of illness under ultrasound guidance by an experienced operator. Pleural fluid analysis showed a neutrophilic effusion with a total white cell count of 900 per mm³ (95% polymorphs, 5% lymphocytes). The pleural fluid analysis findings are summarised in Table 1.

**Table 1:** Pleural fluid analysis findings The pleural fluid protein >3.5 g/dL indicated an exudative effusion; Light's criteria are shown for completeness.

Parameter	Patient value	Reference range
Pleural fluid: pH	7.6	7.60-7.64
Pleural fluid: glucose	33 mg/dL	>60 mg/dL
Pleural fluid: protein	5.12 g/dL	<2.0 g/dL
Serum protein	6.05 g/dL	6.4-8.0 g/dL
Pleural fluid: serum protein ratio	0.85	<0.5
Pleural fluid: lactate dehydrogenase (LDH)	1419 U/L	<200 U/L
Serum LDH	199 U/L	225-450 U/L
Pleural fluid: serum LDH ratio	7.13	<0.6
Pleural fluid: adenosine deaminase (ADA)	23.2 U/L	<40 U/L

The raised pleural fluid protein level of 5.12 g/dL (<2.0 g/dL) classified this as an exudative effusion. Additionally, the fluid analysis fulfilled all three Light's criteria (protein ratio 0.85, LDH 1419 U/L, and LDH ratio 7.13) for an exudative effusion. The pleural fluid for staining for acid-fast bacilli was negative, and the pleural fluid cytology revealed no malignant cells. Pleural fluid culture isolated *Acinetobacter baumannii*, which was sensitive to amikacin, ciprofloxacin, gentamycin, and cefoperazone-sulbactam, but resistant to cefuroxime, co-amoxiclav, and meropenem. The organism demonstrated resistance to meropenem, the carbapenem used for empirical treatment. Blood cultures were negative.

Due to the unavailability of intravenous meropenem at the tertiary care facility, treatment was changed from meropenem to piperacillin-tazobactam 4.5 g every eight hours on the eleventh day of illness. An ultrasound-guided 12 Fr gauge pigtail catheter was also inserted on the same day to drain the complex pleural effusion. With confirmation of a pleural culture positive for *Acinetobacter baumannii *on the twelfth day of illness, intravenous ciprofloxacin 400 mg twice daily was added to the regimen, and piperacillin-tazobactam was continued, as the patient demonstrated clinical improvement.

**Figure 3:**
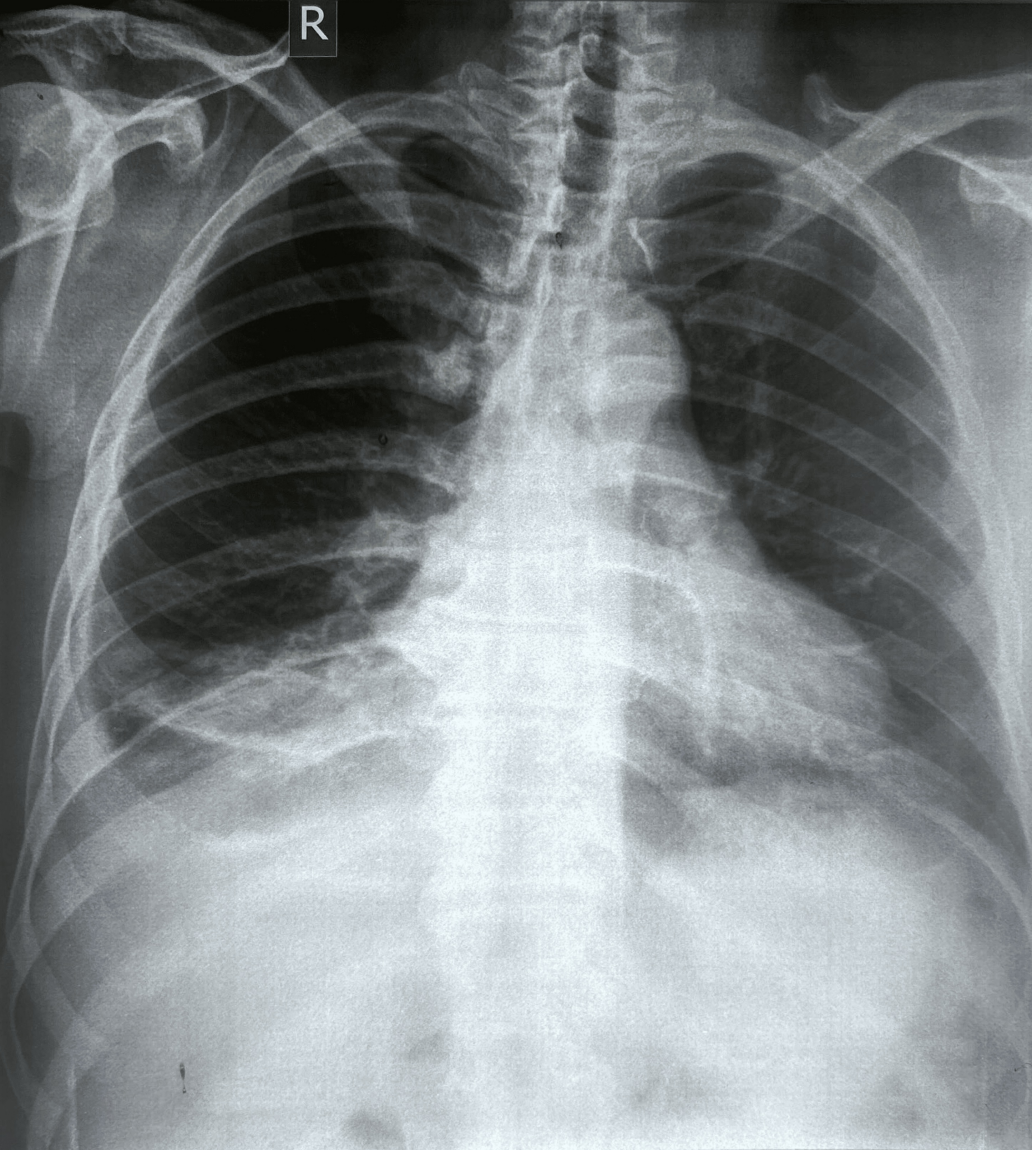
Follow-up Chest X-ray shows a marked interval improvement

The patient received intravenous piperacillin-tazobactam for 19 days, ciprofloxacin for 12 days, along with incentive spirometry exercises and chest physiotherapy. The pigtail catheter was left in situ for 19 days. Clinical stability was achieved with resolution of fever, normalisation of inflammatory markers, and progressive decrease in drain output. The catheter was removed when drainage decreased to less than 20 mL per day, well below the standard threshold of 100 mL per day recommended for drain removal, as it was a complex pleural effusion. Following removal of the pigtail catheter, chest radiography confirmed resolution of the pleural effusion. The patient was discharged with oral ciprofloxacin 500 mg twice daily and continued supportive care for a fortnight, and was reassessed four weeks later with a repeat chest radiograph, which showed a marked interval improvement (Figure 3). Table 2 provides an overview of the case presentation timeline.

**Table 2:** Case presentation timeline N/A - not available; IV - Intravenous; CECT - contrast enhanced computer tomography; CXR - chest X-ray; CRP - C-reactive protein

Day of illness	Clinical event	Temperature (°C)	CRP (mg/L)	Radiographic findings	Intervention
Day 1	Symptom onset	39.2	N/A	N/A	None
Day 4	Local hospital presentation	38.9	418	Right lower zone consolidation + mild effusion	IV meropenem started
Day 7	Transfer to tertiary care	39.6	272	Large right pleural effusion	Continue meropenem
Day 10	Pleural fluid aspiration	39.4	220	CECT chest: septated effusion	Diagnostic aspiration
Day 11	Antibiotic change	39.2	198	N/A	Piperacillin-tazobactam + pigtail insertion
Day 12	Culture confirmed A. baumannii	39.1	185	N/A	Ciprofloxacin added
Day 14	Defervescence	37.4	142	N/A	Continue therapy
Day 18	Clinical improvement	36.8	68	N/A	Continue therapy
Day 25	Near resolution	36.6	32	N/A	Continue drainage
Day 30	Pigtail removal	36.7	18	Resolving effusion (CXR)	Catheter removed
Day 31	Discharge	36.5	10	N/A	Oral ciprofloxacin
Day 45 (week 6)	Follow-up	36.6	6.6	Marked interval improvement (CXR)	Treatment completed

## Discussion

Community-acquired *Acinetobacter *pneumonia is rare, accounting for less than 1% of all community-acquired pneumonia cases in most regions, though higher rates (up to 3-8%) have been reported in tropical areas of Southeast Asia and Northern Australia [5,8]. Pleural effusion as a complication occurs in approximately 20-40% of community-acquired *Acinetobacter *pneumonia cases, with complex parapneumonic effusions or empyema developing in a subset of these patients [7].

This case describes a previously healthy male who developed severe, rapidly progressive right-sided pleuropneumonia with complex septate effusion due to community-acquired *Acinetobacter baumannii* with carbapenem resistance, an uncommon pathogen in community-acquired pneumonia complicated by empyema. Notably, the pleural fluid exhibited exudative characteristics with a predominance of neutrophils, low glucose levels, and markedly elevated LDH levels, consistent with a complex parapneumonic effusion. The pleural fluid pH of 7.6 was unexpectedly high for a complex parapneumonic effusion, where pH values are typically <7.3 and often <7.2 in frank empyema. This atypical finding is difficult to explain but may be attributable to sampling delay, technical factors during measurement (such as air exposure or prolonged time to analysis), or possibly loculation with altered local metabolic characteristics. 

The identified risk factors for community-acquired *Acinetobacter* infections include alcoholism, diabetes mellitus, smoking, chronic renal disease, and chronic lung disease [5]. Notably, our patient had none of these predisposing factors, making this case particularly unusual. Unlike hospital-acquired *Acinetobacter* strains, which frequently exhibit multi-drug resistance (MDR) to multiple antibiotic classes, community-acquired isolates often retain susceptibility to several antimicrobial agents [5,8]. The isolate from our patient remained sensitive to aminoglycosides (amikacin, gentamicin), fluoroquinolones (ciprofloxacin), and cefoperazone-sulbactam, but was resistant to meropenem. This resistance pattern is atypical for community-acquired *Acinetobacter* infections and raises important considerations about the emerging resistance in community settings. While the three failed aspiration attempts raise theoretical concerns about iatrogenic introduction of organisms, the clinical presentation with fever and radiographic consolidation preceding any procedures, along with the carbapenem-resistant pattern matching the treatment failure, strongly supports community acquisition rather than procedural contamination.

The failure of empirical meropenem therapy can be attributed to meropenem resistance in the isolate, highlighting the critical importance of culture-guided therapy even for community-acquired infections. This case underscores that carbapenem resistance can occur in community-acquired *Acinetobacter* strains, challenging the traditional assumption that such resistance patterns are confined to nosocomial infections. The increasing prevalence of antimicrobial resistance in South Asia [9] may contribute to the emergence of resistant community-acquired isolates in this region.

This case underscores the importance of incorporating local antibiogram data into empirical therapy decisions. The emergence of carbapenem resistance in our community-acquired Acinetobacter isolate challenges traditional assumptions that such resistance patterns are confined to nosocomial infections. In South Asia, where antimicrobial resistance rates are rising [9], empirical therapy guidelines for severe community-acquired pneumonia may need to consider broader coverage, particularly for patients with complicated presentations such as pleural effusion. Early microbiological sampling, close monitoring of clinical response, and prompt adjustment based on culture results and local resistance patterns are essential. This case supports the need for enhanced regional surveillance and institutional antimicrobial stewardship programs that regularly review and incorporate local antibiogram data into treatment protocols.

Our patient's pleural fluid analysis showed low glucose levels (33 mg/dL) and elevated LDH (1419 U/L) with an LDH ratio of 7.13, clearly indicating a complex parapneumonic effusion warranting intrapleural catheter drainage according to established guidelines [10]. The combination of appropriate drainage and antimicrobial therapy was essential for successful management. The isolation of *Coliform* species from sputum cultures was considered likely oropharyngeal contamination given the low pus cell count (10-25 cells per low-power field) and the presence of epithelial cells, which are markers of upper airway contamination [11].

The treatment approach in this case required modification based on local antibiotic availability and the culture results. While sulbactam-containing regimens (ampicillin-sulbactam or cefoperazone-sulbactam) are preferred for *Acinetobacter* infections with carbapenem resistance [12], piperacillin-tazobactam was used due to the unavailability of these agents. The clinical improvement observed with piperacillin-tazobactam, combined with ciprofloxacin (to which the organism was sensitive) and appropriate drainage, suggests that the combination therapy and procedural intervention were effective. However, tazobactam has limited activity against *Acinetobacter* species compared to sulbactam, and the response may have been primarily driven by the ciprofloxacin component and catheter drainage rather than the β-lactam agent.

Compared to previous reports of community-acquired *Acinetobacter* pneumonia, our case demonstrated several noteworthy features. While Chen et al. noted that community-acquired *Acinetobacter baumannii *pneumonia typically presents with fulminant, rapidly progressive respiratory failure, often accompanied by high fever, severe hypoxemia, and early development of complications such as pleural effusion, bacteremia, and septic shock [13], our patient remained hemodynamically stable throughout his illness. However, the clinical deterioration despite three days of meropenem treatment, complicated by a complex parapneumonic pleural effusion, is consistent with this pathogen's virulence and underscores the importance of selecting appropriate antimicrobials based on susceptibility patterns.

The primary strength of this case lies in the prompt collection of pleural fluid for culture under ultrasound guidance, which enabled rapid identification of pathogens and susceptibility-guided therapy. However, several limitations warrant discussion. First, the complete characterisation of carbapenem resistance was hindered by using only meropenem rather than multiple carbapenems, and by the lack of minimum inhibitory concentration (MIC) values. This prevents the isolate from being definitively classified as carbapenem-resistant *Acinetobacter baumannii *according to standard criteria. Second, the initial empirical treatment did not include coverage for atypical pathogens or consideration of local resistance patterns. Third, preferred sulbactam-containing agents were unavailable in the public hospital setting, necessitating the use of alternative regimens. Fourth, the initial attempts at pleural drainage should have been performed under ultrasound guidance from the outset to improve success rates and minimise patient discomfort. Ultrasound imaging was used for procedural guidance, but static images were not archived for documentation, which limits our ability to demonstrate the sonographic characteristics of the septated effusion. However, CT imaging confirmed the complex septated nature of the collection.

## Conclusions

This case demonstrates that community-acquired *Acinetobacter baumannii* can cause severe, complex parapneumonic effusion even in immunocompetent individuals without traditional risk factors. The emergence of carbapenem resistance in community isolates, as demonstrated in our patient, represents an evolving threat that challenges conventional treatment approaches.
